# Scientific Items

**Published:** 1884-02-20

**Authors:** 


### SCIENTIFIC ITEMS.

**Influence of Science on Religion.**—Herbert Spencer in the Popular Science Monthly for January :

While the beliefs to which analytic science leads are such as do not destroy the object-matter of religion, but simply transfigure it, science under its concrete forms enlarges the sphere for religious sentiment. From the very beginning the progress of knowledge has been accompanied by an increasing capacity for wonder. Among savages, the lowest are the least surprised when shown remarkable products of civilized art, astonishing the traveler by their indifference. And so little of the marvelous do they perceive in the grandest phenomena of Nature that any inquiries concerning them they regard as childish trifling. This contrast in mental attitude between the lowest human beings and the higher human beings around us is paralleled by the contrasts among the grades of these higher human beings themselves. It is not the rustic, nor the artisan, nor the trader, who sees something more than a mere matter of course in the hatching of a chick ; but it is the biologist, who, pushing to the uttermost his analysis of vital phenomena, reaches his greatest perplexity when a speck of protoplasm under the microscope shows him life in its simplest form, and makes him feel that however he formulates its processes the actual play of forces remains unimaginable. Neither in the ordinary tourist nor in the deer-stalker climbing the mountains above him, does a Highland glen rouse ideas beyond those of sport or of the picturesque ; but it may, and often does, in the geologist. He, observing that the glacier-rounded rock he sits on has lost by weathering, but half an inch of its surface since a time far more remote than the beginning of human civilization, and then trying to conceive the slow denudation which has cut out the whole valley, has thoughts of time and of power to which they are strangers—thoughts which, already utterly inadequate to their objects, he feels to be still more futile on noting the contorted beds of gneiss around, which tell him of a time, immeasurably more remote, when far beneath the earth's surface they were in a half-melted state, and again tell him of a time, immensely exceeding this in remoteness, when their components were sand and mud, on the shores of an ancient sea. Nor is it in the primitive peoples who supposed that the heavens rested on the mountain-tops, any more than in the modern inheritors of their cosmogony who repeat that "the heavens declare the glory of God," that we find the largest conceptions of the universe or the greatest amount of wonder excited by contemplation of it. Rather, it is in the astronomer, who sees in the sun a mass so vast that even into one of his spots our earth might be plunged without touching its edges ; and who by every finer telescope is shown an increased multitude of such suns, many of them far larger.—*Med. Age.*

**The** strange condition of the sun, says the Graphic, at certain periods of the day excited great interest in Southern India early in September. Both morning and evening the sun was tinged with a bluish-yellow color, and its disc became bluish-green, suggesting to jokers the old childish story that the moon was made of green cheese, and that the sun was of apparently similar composition. It is believed that the phenomenon was due to the passage across India of a tremendous volume of sulphurous vapor arising from the volcanic disturbances of Sunda Straits. It is worth noting that, about the same time, in Port-of-Spain, Trinidad, the sun toward evening became blue.—*Mechanical News.*

**The** approaches to the St. Gothard Tunnel are thought to be about as wonderful as the great tunnel itself. To get up to the level of the tunnel the railway track makes many spirals, winding, in some instances, three times around a single mountain, on three terraces, one above the other, through twisting tunnels. The curves are, however, so gradual as to be hardly noticeable unless one carries a compass. Then is seen the curious fact that the needle makes complete circuits, and is constantly shifting its position.—*Mechanical News.*

**In** his report to the Secretary of the Interior, Mr. J. W. Powell, director of the United States Geological Survey, gives some interesting facts. In Colorado, valuable beds of anthracite and of bituminous coal have been found, surpassing in quality any heretofore discovered in that region, and indications of large deposits of iron are visible. Evidences of the former existence of a large fresh water lake in western Nevada have been discovered. Traces of a vast continental glacier have been found, of so well defined a character as possibly to change the present geological conclusions of previous explorations. In the work done is included a survey of the Cascade range in Oregon and Northern California. Mr. Powell says that this region is, perhaps, the holder of the grandest and most extensive display of natural phenomena in the world, and its exploration and thorough investigation will add greatly to the facts of geological science.—*Mechanical News.*

**Easy Writing.**—According to the Lancet, "brain tension is not a proof of strength, but of weakness. The knit brow, straining eyes, and fixed attention of the scholar are not tokens of power, but of effort. The intellectual man with a strong mind does his brain work easily. Tension is friction, and the moment the toil of a growing brain becomes laborious it should cease. We are unfortunately so accustomed to see brain work done with an effort that we have come to associate effort with work, and to regard tension as something tolerable, if not natural. As a matter of fact, no man should ever knit his brow as he thinks, or in any way evince effort as he works. The best brain work is done easily, with calm spirit, an equable temper, and in jaunty mood. All else is the toil of a weak or ill-developed brain straining to accomplish a task which is relatively too great for it."—*Ex.*

